# Application of Acute Physical Therapy in Respiratory Failure Secondary to Bilateral Medial Medullary Infarction and Long-Term Follow-Up: A Case Report

**DOI:** 10.7759/cureus.112484

**Published:** 2026-07-11

**Authors:** Xiaojuan Qian, Yongcai Shui, Ting Zhang, Liqiong Guo, Yaqin Lu

**Affiliations:** 1 Rehabilitation Medicine, Gansu Provincial Maternal and Child Health Hospital (Gansu Provincial Central Hospital), Lanzhou City, CHN

**Keywords:** bilateral medial medullary infarction, dysphagia, quadriplegia, respiratory care, respiratory disorders

## Abstract

Bilateral medial bulbar infarction (BMMI) represents a rare yet critical form of posterior circulation ischemic stroke encountered in clinical practice. The condition progresses rapidly and is associated with a heightened risk of secondary central respiratory failure, severe pulmonary infection, and multiple systemic functional impairments, including dysphagia, limb paralysis, and sensory abnormalities. Consequently, both the disability and mortality rates remain elevated. Currently, systematic clinical studies focusing on early physical intervention, phased rehabilitation programs, and long-term prognosis follow-up for patients with BMMI during the acute phase are relatively limited. This report reviews the clinical diagnosis and treatment, early acute physical therapy, and two-year long-term rehabilitation follow-up data of a 53-year-old male patient with BMMI, who presented with acute central respiratory failure, severe pulmonary infection, and pronounced bulbar palsy. It summarizes the clinical progression characteristics, stepwise rehabilitation intervention strategies, and prognostic patterns associated with this condition. The objective is to provide a clinical reference for early rehabilitation interventions, the formulation of individualized treatment plans, and the assessment of long-term prognosis for patients with BMMI complicated by severe multi-system dysfunction.

## Introduction

Medial medullary infarction (MMI) is a rare ischemic cerebrovascular disorder of the posterior circulation, accounting for 0.5-1.5% of all ischemic strokes [1]. Bilateral medial medullary infarction (BMMI) is an even rarer subtype of MMI. This condition selectively affects the core functional regions of the medulla oblongata, potentially damaging the brainstem respiratory regulatory center, descending motor conduction tracts, and swallowing nerve pathways. Typical clinical manifestations include quadriplegia, dysarthria, severe dysphagia, and impaired superficial and deep sensation. Critically ill patients may experience sudden central respiratory rhythm disorders and ventilatory failure, which pose significant clinical risks and result in poor long-term prognosis [2,3].

Radiologically, bilateral paramedian medullary hyperintensities, separated by the midline raphe on axial DWI sequences, create the characteristic “heart sign,” a pathognomonic MRI marker for BMMI that aids in rapid clinical diagnosis [4]. The acute phase, occurring within several weeks following a stroke, represents a critical window for neural plasticity and central nervous system repair. Increasing evidence indicates that initiating shorter and more frequent treatments within the first 24-48 hours post stroke is safe. During this period, the neural tissue surrounding the lesion exhibits enhanced synaptogenesis and axonal budding. Rehabilitation can provide consistent peripheral afference, thereby promoting the structural and functional reorganization of surviving neural circuits around the ischemic core [5].

Given the exceedingly low incidence of BMMI, current clinical studies primarily focus on its imaging characteristics and short-term diagnostic and therapeutic strategies. Notably, there is a scarcity of case reports addressing systematic early physical rehabilitation interventions and comprehensive long-term follow-up for severe BMMI patients who experience respiratory failure and require invasive mechanical ventilation [6]. In this report, we present the complete diagnosis, treatment, and rehabilitation follow-up of a patient with BMMI complicated by acute respiratory failure, severe pulmonary infection, and intractable dysphagia.

## Case presentation

A 53-year-old male patient presented with a sudden onset of vertigo and slurred speech lasting for six hours. He did not exhibit symptoms such as nausea, vomiting, altered consciousness, limb weakness, or double vision. He was admitted to the neurology department with a diagnosis of acute ischemic cerebrovascular disease.

Upon admission, a physical examination revealed a blood pressure of 158/89 mmHg. The patient was alert but displayed dysarthria. Neurological examination confirmed cranial nerve dysfunction involving the hypoglossal nerve, along with functional impairment of the medullary pathways of the glossopharyngeal and vagus nerves. This resulted in typical central bulbar palsy, characterized by dysarthria, oropharyngeal dysphagia, a weakened gag reflex, and insufficient voluntary cough strength; no abnormalities of other cranial nerves were noted. Limb muscle strength and tone were normal; however, his movements were marked by instability and inaccuracy. Bilateral limb coordination was impaired. The finger-to-nose test revealed bilateral dysmetria, accompanied by a pronounced intention tremor and inaccurate target pointing. The heel-to-shin test demonstrated jerky sliding motion, frequent deviation of the heel away from the tibia, and poor movement precision.

**Figure 1:**
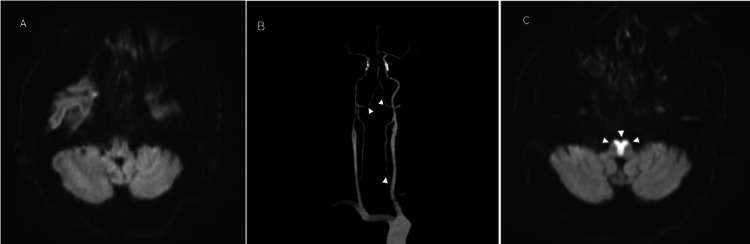
Imaging findings on Day 1 and Day 2 (A) DWI-MRI on Day 1 showing no acute ischemic lesion observed in the medulla oblongata (early DWI-negative window); (B) Head and neck CTA showing severe vertebral artery stenosis (arrowheads); (C) DWI-MRI on Day 2 showing bilateral paramedian medullary infarcts (arrowheads) presenting the classic BMMI “heart sign” imaging feature. MRI: magnetic resonance imaging; DWI: diffusion-weighted imaging; CTA: computed tomography angiography

Neurological function assessment yielded a National Institutes of Health Stroke Scale (NIHSS) [7] score of 5, a modified Rankin scale (mRS) [8] score of 3, and a Kubota Water Swallowing Test [9] grade of 2. Auxiliary examinations did not reveal any clear acute ischemic lesions on the head MRI (Figure 1A), while the CT angiography (CTA) of the head and neck vessels indicated multiple severe stenoses of the vertebral arteries (Figure 1B).

Following admission, the patient's condition progressively deteriorated. By Day 2, his articulation had significantly worsened, and he developed new left-limb hemiplegia. Re-examination revealed an NIHSS score of 9, an mRS score of 5, and a Kubota Water Swallowing Test grade of 3. A re-evaluation of the head MRI showed acute ischemic lesions bilaterally in the medial medulla oblongata, confirming a diagnosis of acute cerebral infarction in that region (Figure 1C).

On Day 3, the patient's condition deteriorated significantly, marked by weak coughing and respiratory distress. Auscultation of the lungs revealed wet rales, and a concurrent pulmonary infection was identified. An urgent arterial blood gas analysis indicated an arterial partial pressure of oxygen (PaO_2_) of 56 mmHg and a partial pressure of carbon dioxide (PCO_2_) of 31 mmHg, resulting in a diagnosis of acute hypoxemic respiratory failure. Tracheal intubation and invasive ventilator-assisted ventilation were performed immediately, along with the insertion of a gastric tube and urinary catheter. Invasive ventilation was initiated using pressure-controlled ventilation mode, with a fraction of inspired oxygen (FiO₂) of 60%, positive end-expiratory pressure (PEEP) of 8 cmH₂O, tidal volume of 450 mL, and a respiratory rate of 18 breaths per minute. Simultaneously, comprehensive multi-organ supportive treatments were administered to stabilize vital signs: intravenous antibiotics were provided for the pulmonary infection, mucolytics and physical sputum drainage were employed to facilitate expectoration, inhaled bronchodilators alleviated bronchospasm, early enteral tube feeding ensured nutritional supplementation, and targeted interventions, including rational fluid management, stress ulcer prophylaxis, and avoidance of hepatorenal toxic drugs, were implemented for multi-organ protection.

By Day 4, the patient's vital signs had stabilized, and there were no contraindications to initiating rehabilitation interventions. Early rehabilitation commenced, accompanied by a comprehensive baseline assessment: Glasgow Coma Scale (GCS) score E3VTM4, which measures consciousness impairment; Richmond Agitation-Sedation Scale (RASS) [10] score -3, indicating a moderately sedated state; and ICU Mobility Scale (IMS) [11] score 0, denoting a complete inability to perform any spontaneous limb movement in bed. The patient required invasive ventilator-assisted ventilation, characterized by abnormal thoracic breathing as the predominant respiratory pattern. The Modified Barthel Index (MBI) score [12] was 0, reflecting complete dependence for all activities of daily living. The risks for pressure ulcers, thrombosis, and nutritional deficits were all classified as high. The Caprini Venous Thromboembolism Risk Score [13] was 6 points for stratifying thrombosis risk, the Nutritional Risk Screening 2002 score [14] was 6 points for malnutrition screening, and the Braden Pressure Injury Risk Scale [15] score was 8 points, indicating an extremely high risk for pressure ulcers.

On Day 6, the patient's sedation was reduced, consciousness was clear, and partial spontaneous breathing was restored. Following the complication prevention training, a stepwise active rehabilitation program was initiated. Early rehabilitation aimed to prevent severe complications, safeguard joint range of motion, delay disuse atrophy of respiratory and skeletal muscles, and enhance pulmonary ventilation. This encompassed proper limb positioning, patient turning, and back tapping every two hours, alongside full-range passive joint movement training for the limbs. Simultaneously, it included postural drainage, correction of breathing patterns, and external diaphragm pacemaker therapy (Model HLO-CJ13A (Hanfei Medical, Guangdong, China) with pulse frequency 40 Hz, pulse width 200 μs, pacing rate nine cycles/minute, titrated intensity 8-14 units, 30 minutes per session twice daily; bilateral electrodes attached to the inferolateral one-third of the sternocleidomastoid muscle) to facilitate airway secretion drainage and mitigate pulmonary inflammation. This program improved the breathing pattern and enhanced respiratory muscle strength through abdominal breathing, pursed-lip breathing, and respiratory muscle resistance training (threshold resistance load starting at 5 cmH₂O, with a progressive increment of 2 cmH₂O weekly, for 20 min twice daily). Additionally, active limb training was performed. The tracheal cannula was successfully removed on Day 13.

On Day 17, the patient was transferred to a general ward. A videofluoroscopic swallowing study (VFSS) esophagography indicated dysphagia during the oral and pharyngeal phases, with a Penetration-Aspiration Scale (PAS) [16] grade of 1 and no aspiration observed during swallowing; the cricopharyngeal muscle exhibited normal opening function. The patient's active forced cough was assessed as grade 2 according to the standardized five-point voluntary cough strength grading system (Grade 1 = no effective cough; Grade 2 = weak cough with insufficient expiratory flow leading to obvious sputum retention and poor secretion clearance; Grade 3 = moderate effective cough; Grade 4 = strong effective cough; Grade 5 = forceful explosive cough). Prior to respiratory rehabilitation on Day 17, the patient demonstrated an ineffective cough rated nearly Grade 1, characterized by severe sputum accumulation and fluctuating peripheral oxygen saturation. Following standardized airway clearance and respiratory muscle training, cough strength improved to Grade 2; however, expectoration remained inadequate, necessitating continuous oxygen supplementation.

Rehabilitation program

A comprehensive rehabilitation intervention was implemented, which included progressive breathing exercises and airway clearance training to enhance cardiopulmonary function and expectoration capacity. Additionally, training was given for the oropharyngeal muscle group, and combined swallowing neuromuscular electrical stimulation (NMES) (Model HB610B; Haobo Medical Co., Ltd., Suzhou, Jiangsu, China) was employed to improve swallowing function. The stimulation settings were fixed at a pulse frequency of 80 Hz and a pulse width of 300 μs, with an adjustable output current ranging from 0 to 25 mA, titrated to individual tolerance; each treatment session lasted 20 minutes and occurred once daily. Two pairs of sponge electrodes were bilaterally attached over the suprahyoid and infrahyoid swallowing muscle groups to activate the oropharyngeal motor pathway synchronously. Furthermore, active limb movement training, sitting balance training, somatic sensory input training, and limb coordination training were incorporated, guided by standardized coordination test results.

**Figure 2:**
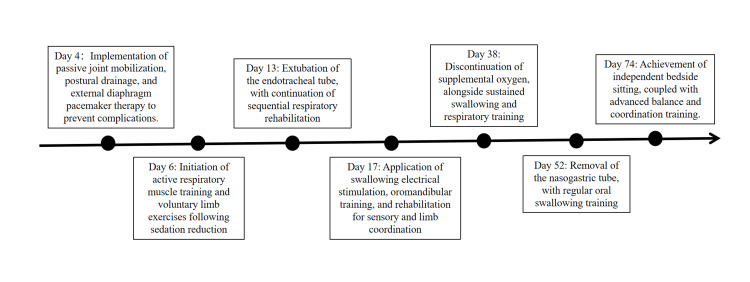
Progress in rehabilitation treatment

On Day 38, the patient was completely weaned off oxygen therapy, and the nasogastric tube was removed on Day 52. By Day 74, the patient was able to sit independently at the bedside but still required assistance for turning, sitting up, and transferring between the bed and chair. The patient's ability to perform activities of daily living improved, as indicated by an MBI score of 48. Following discharge, we developed an individualized home rehabilitation plan along with a structured outpatient follow-up schedule. The progress of rehabilitation treatment is shown in Figure 2.

At the two-year follow-up, the patient's vital signs remained stable. The patient was wheelchair-bound and continued to require assistance from one individual for daily activities. Table 1 summarizes the in-hospital assessments and two-year follow-up outcomes.

**Table 1:** Summary of in-hospital assessments and the two-year follow-up outcomes. Kubota Water Swallowing Test: an ordinal 1–5 scale, with higher grades indicating more severe oropharyngeal dysphagia [9]; ICU Mobility Scale (IMS): a 0–10 scale measuring physical mobility in critically ill patients; score 0 represents complete absence of spontaneous active limb movement in supine position, and higher scores denote better mobility performance [11]; MMT grading: 0–5 scale, where grade 5 indicates full normal muscle strength. Explanation of MBI score decline from Day 2 to Day 4: The patient developed acute central respiratory failure on Day 3 and received invasive mechanical ventilation, resulting in complete loss of self-care ability.

Assessment Item	On Admission	Day 2 after Admission	Day 4 after Admission (Rehabilitation Baseline)	Day 6 after Admission	Day 13 after Admission	Day 38 after Admission	Day 52 after Admission	Day 74 after Admission	2-Year Follow-Up
Glasgow Coma Scale (GCS)	15 (Alert)	13 (Somnolent)	Sedated	Sedated	15 (Alert)	15 (Alert)	15 (Alert)	15 (Alert)	15 (Alert)
Richmond Agitation-Sedation Scale (RASS) [10]	0 (Alert and Calm)	-1 (Light Sedation)	-3 (Moderate Sedation)	0 (Light Sedation)	0 (Alert and Calm)	0 (Alert and Calm)	0 (Alert and Calm)	0 (Alert and Calm)	0 (Alert and Calm)
National Institutes of Health Stroke Scale (NIHSS) [7]	5	9	-	-	8	7	7	5	5
modified Rankin Scale (mRS) [8]	3	5	-	-	4	4	4	4	4
Manual Muscle Testing (MMT): biceps brachii, triceps brachii, Iliopsoas, quadriceps femoris, tibialis anterior (Right/Left)	Normal	5/3，5/2， 4/2，5/3，4/3	-	-	5/3，5/3， 4/2，5/3，4/4	5/3，5/3， 4/4，5/3，4/4	5/3，5/4， 4/4，5/3，4/4	5/3，5/4， 4/4，5/4，4/4	5/4，5/4， 4/4，5/4，4/4
Kubota Water Swallowing Test [9]	Grade 2	Grade 3	-	-	Grade 2	Grade 3	Grade 2	Grade 2	Grade 2
Modified Barthel Index (MBI) [12]	90	46	0	0	24	35	45	48	48
Caprini Venous Thromboembolism Risk Score [13]	4 (Moderate Risk)	6 (High Risk)	8 (Very High Risk)	8 (Very High Risk)	6 (High Risk)	4 (Moderate Risk)	4 (Moderate Risk)	4 (Moderate Risk)	4 (Moderate Risk)
Braden Pressure Injury Risk Scale [15]	18 (No Risk)	16 (Low Risk)	8 (High Risk)	12 (High Risk)	16 (Low Risk)	16 (Low Risk))	16 (Low Risk)	16 (Low Risk)	16 (Low Risk)
ICU Mobility Scale (IMS) [11]	8	2	0	0	2	5	5	6	7

## Discussion

BMMI is a rare type of posterior circulation stroke with a markedly poor prognosis, accounting for less than 1% of all acute strokes. The primary pathological damage involves the disruption of the brainstem respiratory regulatory network, the interruption of the downward respiratory drive pathway, and injury to the swallowing and motor nerve pathways. These factors lead to multiple functional deficits, including central ventilation dysfunction, quadriplegia, and severe bulbar palsy. Complications such as pulmonary infection and sputum retention contribute to persistently elevated disability and mortality rates associated with this condition [17]. Consequently, elucidating the pathological mechanisms underlying respiratory failure secondary to BMMI and leveraging opportunities for early rehabilitation intervention are of substantial clinical importance for halting disease progression, reducing mortality rates, and improving the long-term prognosis for patients [18].

Central respiratory failure constitutes the most critical early complication of BMMI, with its primary pathogenesis stemming from organic damage to the respiratory regulatory structures within the medulla oblongata. The medullary reticular formation is responsible for generating respiratory rhythm and transmitting ventilatory signals to peripheral respiratory motor neurons. Bilateral medial medullary infarction disrupts bilateral descending respiratory conduction tracts and impairs respiratory chemical reflex neurons, leading to the abolition of spontaneous ventilatory drive and resulting in postural drainage, external diaphragmatic and limb placement, and passive joint movement, resulting in central hypoventilation [19-24]. Additionally, concomitant weakness of the hypoglossal and bulbar muscles compromises airway clearance, further precipitating refractory aspiration pneumonia [25].

Hu et al.'s cohort study involving 11 patients with BMMI reported an unfavorable prognosis rate of 81.8% [22]. Among these patients, six developed severe pulmonary infections, and only a few regained the ability for independent self-care. This is mirrored by the clinical trajectory of the current patient; specifically, early severe sputum retention swiftly escalated to respiratory failure, necessitating emergency intubation. This observation underscores pulmonary infection as an independent risk factor for poor prognosis in acute BMMI.

The theory of neural plasticity suggests that the initial period following a stroke constitutes a critical window for the repair of neural functions. Early implementation of standardized and multidimensional rehabilitation interventions can significantly reduce the risk of severe disuse injuries and promote the remodeling of damaged neural pathways, which is vital for enhancing the prognosis of patients who have experienced severe strokes [26,27]. In this instance, acute physical therapy began on the fourth day after onset, focusing on complication prevention during the critical care intervention phase. Physical interventions, which included standardized, proper pacing, effectively prevented severe complications such as pressure ulcers, deep vein thrombosis in the lower extremities, joint ankylosis, and disuse atrophy of the respiratory muscles. Respiratory rehabilitation therapy primarily stimulates diaphragm function, improves pulmonary ventilation reserve, aids in the clearance of airway secretions, and accelerates the resolution of pulmonary inflammation. The patient successfully underwent extubation within 13 days of invasive ventilation, a result attributed to the early initiation of acute multidisciplinary physiotherapy. Compared with the ventilation duration reported in Hu et al.’s 2022 BMMI cohort [22], the current patient exhibited a relatively shortened ventilator dependency period, which may be partially attributed to timely staged respiratory rehabilitation, young age, and absence of chronic pre-existing respiratory comorbidities. This observation underscores the essential role of acute physical therapy in the early management of severe BMMI patients.

During the functional recovery phase, the stepwise and individualized comprehensive rehabilitation program systematically and efficiently restored multiple system functions, including respiration, swallowing, and limb mobility. Respiratory rehabilitation, which encompasses the progressive correction of breathing patterns, resistance training for respiratory muscles, and airway clearance interventions, facilitates the gradual restoration of stable autonomous ventilation and effective expectoration in patients transitioning from complete ventilator dependence and an inability to expectorate independently. In the present case, after 37 days from the onset of his condition, the patient was completely free from oxygen therapy support, and his respiratory function largely returned to normal. Swallowing rehabilitation specifically targets patients experiencing mixed swallowing disorders during the oral and pharyngeal stages. This approach employs a precise intervention plan that integrates muscle group training, reflex induction, and physical electrical stimulation, effectively addressing the swallowing function impairment associated with bulbar palsy.

The gastric tube was successfully removed 52 days after the onset of the disease, allowing for the resumption of oral feeding and completely preventing complications such as infection, nutritional imbalance, and esophageal mucosal damage related to prolonged gastric tube indwelling [28]. Observations at the two-year follow-up indicated that the neural pathway damage resulting from ischemic necrosis in the core area of the brainstem was irreversible. The patient continues to exhibit mild swallowing abnormalities and diminished limb muscle strength over the long term, maintaining significant dependence on daily activities. He remains unable to independently perform tasks such as turning over and transferring between beds and chairs, necessitating ongoing dedicated care.

## Conclusions

Patients experiencing acute respiratory failure due to BMMI face a perilous condition characterized by rapid progression, significant functional impairment across multiple systems, various complications, and a high rate of long-term disability. Clinically, it is essential to detect changes in the patient's condition at an early stage, promptly address respiratory failure, manage pulmonary infections, and capitalize on the rehabilitation window to implement a stepwise and multidimensional acute physical rehabilitation intervention as soon as possible. This approach aims to prevent severe complications, support favorable clinical trajectories associated with shorter invasive ventilation duration, and facilitate the recovery of respiratory, swallowing, and limb functions. Follow-up results over two years confirmed that standardized full-course rehabilitation could stabilize a patient's vital signs, although it did not restore the basic self-care abilities.
